# Butaphosphan Effects on Glucose Metabolism Involve Insulin Signaling and Depends on Nutritional Plan

**DOI:** 10.3390/nu12061856

**Published:** 2020-06-22

**Authors:** Maria Amélia Agnes Weiller, Joao Alveiro Alvarado-Rincón, Carolina Bespalhok Jacometo, Carlos Castilho Barros, Izabel Cristina Custódio de Souza, Lucas Teixeira Hax, Thaís Casarin da Silva, Patrícia Mattei, Antônio Amaral Barbosa, Josiane de Oliveira Feijó, Rubens Alves Pereira, Cassio Cassal Brauner, Viviane Rohrig Rabassa, Francisco Augusto Burkert Del Pino, Marcio Nunes Corrêa

**Affiliations:** 1 Núcleo de Pesquisa, Ensino e Extensão em Pecuária—NUPEEC, Faculdade de Veterinária, Universidade Federal de Pelotas, Pelotas RS 96160-000, Brazil; mariaamelia.weiller86@gmail.com (M.A.A.W.); joaoal13@hotmail.com (J.A.A.-R.); cbespalhok@unisalle.edu.co (C.B.J.); lucashax@gmail.com (L.T.H.); thais.casarindasilva@gmail.com (T.C.d.S.); patymattei@gmail.com (P.M.); antoniobarbosa.vet@hotmail.com (A.A.B.); josianeofeijo@gmail.com (J.d.O.F.); rubens@ibasa.com (R.A.P.); cassiocb@gmail.com (C.C.B.); vivianerabassa@gmail.com (V.R.R.); fabdelpino@gmail.com (F.A.B.D.P.); 2 Programa de Zootecnia, Facultad de Ciencias Agropecuarias, Universidad de La Salle, Bogota DC 110231, Colombia; 3 Faculdade de Nutrição, Universidade Federal de Pelotas, Pelotas, RS 96010-610, Brazil; barros_cc@yahoo.com.br; 4 Instituto de Biologia, Universidade Federal de Pelotas, Pelotas RS 96160-000, Brazil; belcustodio20@yahoo.com.br; 5 Laboratório Ibasa, Porto Alegre RS 90220-030, Brazil

**Keywords:** phosphorus, gene expression, gluconeogenesis, metabolism, mice

## Abstract

Butaphosphan is an organic phosphorus compound used in several species for the prevention of rapid catabolic states, however, the mechanism of action remains unclear. This study aimed at determining the effects of butaphosphan on energy metabolism of mice receiving a normal or hypercaloric diet (HCD) and submitted or not to food restriction. Two experiments were conducted: (1) during nine weeks, animals were fed with HCD (*n* = 28) ad libitum, and at the 10th week, were submitted to food restriction and received butaphosphan (*n* = 14) or saline injections (*n* = 14) (twice a day, for seven days) and; (2) during nine weeks, animals were fed with a control diet (*n* = 14) or HCD (*n* = 14) ad libitum, and at the 10th week, all animals were submitted to food restriction and received butaphosphan or saline injections (twice a day, for seven days). In food restriction, butaphosphan preserved epididymal white adipose tissue (WAT) mass, increased glucose, NEFA, and the HOMA index. In mice fed HCD and submitted to food restriction, the butaphosphan preserved epididymal WAT mass. Control diet influences on *PI3K*, *GCK*, and *Irs1* mRNA expression. In conclusion, butaphosphan increased blood glucose and reduced fat mobilization in overweight mice submitted to caloric restriction, and these effects are influenced by diet.

## 1. Introduction

The use of organic minerals in animal nutrition has been stimulated in recent years due to several advantages that these nutrients have compared to their inorganic counterparts. An organic mineral consists of a mineral ion chemically bound to an organic molecule (e.g., amino acids, proteins, and carbon chains), forming a structure with characteristics of higher stability and bioavailability [1]. Otherwise, inorganic minerals are added to diets generally in the form of salts (oxides and sulphates) [2] and can suffer high loss rate during the digestion processes, leading to economic losses and environmental contamination [3,4].

Butaphosphan is an organic phosphorus compound that is available in many countries in combination with cyanocobalamin (vitamin B12) as an injectable veterinary product known as Catosal^®^. Studies with isolated butaphosphan or Catosal^®^ in cattle, ewes, horses, swine, broilers, dogs, and mice have shown that these products improved general health status by stimulating feed intake, improving liver and muscle function, and homeostasis [5,6,7,8,9,10,11]. In cattle, butaphosphan is used specially during transition periods when a state of acute negative energy balance (NEB), largely due to an inadequate dry matter intake (DMI), can lead to mobilization of significant amounts of body fat, an increase blood non-esterified fatty acid (NEFA) concentration, and hepatic lipidosis [5,12,13].

It is believed that butaphosphan and cyanocobalamin association favors the phosphorylation of molecules that intermediate metabolic pathways, such as a gluconeogenesis, glycolysis, and the Krebs cycle, improving the synthesis of the co-nutrients (ATP and ADP mainly) and consequently increasing blood glucose [14]. However, it is still unclear if these results refer mainly to one of the nutrients, butaphosphan or cyanocobalamin.

A study using mice as a model, feeding a diet rich in cholesterol, showed that phosphorus restriction increased hepatic triglycerides, induced to steatosis and reduced the hepatic expression of genes related to cholesterol metabolism and fatty acids biosynthesis [15]. Additionally, as reported in other species, phosphate restriction reduced food consumption in rats, and consequently they lost weight and presented energy deficiency [16]. Evidences suggest that a misbalance in phosphorus metabolism is related with the risk of development and severity of metabolic syndrome in humans [17,18,19], and phosphorus depletion is associated with fulminant hepatic failure, a consequence of severe liver injury [20]. Additionally, dietary phosphorus increased endogenous glucose production by stimulating gluconeogenic and glycogenolysis pathways and may be partially responsible for glucose intolerance and insulin resistance [21,22]. Hasi et al. [6] investigated the effects of butaphosphan solution on endurance capability and energy metabolism in mice and found that butaphosphan increased glycogen, adenosine triphosphate (ATP), and adenosine diphosphate (ADP) in liver and skeletal muscle, indicating that the solution can significantly enhance the energy metabolism in mice by improving liver and muscle glycogen storage and promoting the synthesis of ATP and ADP.

In the present study we hypothesized that butaphosphan effects involve insulin regulation and signaling and depend on energy status. Thus, our aim was to investigate the effects of butaphosphan treatment on the hepatic metabolism and insulin resistance on a model of acute state of energy deficit by feeding male mice with a hypercaloric diet followed by caloric restriction period, inducing to fat mobilization and hepatic challenge. Additionally, we also wanted to elucidate if the effects of butaphosphan treatment in feed restricted mice are dependent on the type of diet which animals have been fed (control or hypercaloric).

## 2. Materials and Methods 

### 2.1. Experimental Design

All procedures were approved by the Animal Care and Use Committee from the Federal University of Pelotas (register number: 6936) and are in accordance with the international guidelines for animal care and use. Ninety-days-old C57BL/6 male mice (Federal University of Pelotas Bioterium, Pelotas, Brazil) were housed in a temperature-controlled room (23 °C), on a 12 h light–dark cycle. The research was based on two experiments, as described below.

#### 2.1.1. Experiment 1

Mice were randomly assigned to four groups: ***HS*** (*n* = 7), fed hypercaloric diet (HCD) ad libitum during 10 weeks and received saline injections during the last week; ***HB*** (*n* = 7), fed HCD ad libitum during 10 weeks and received butaphosphan injections during the last week; ***HRS*** (*n* = 7)*,* fed HCD ad libitum for 9 weeks and during the 10th week were submitted to feed restriction and received saline injections; and ***HRB*** (*n* = 7), fed HCD ad libitum for 9 weeks and during the 10th week were submitted to feed restriction and received butaphosphan injections.

#### 2.1.2. Experiment 2

Mice were randomly assigned to four groups: ***CRS*** (*n* = 7), fed control diet ad libitum during 9 weeks and during the 10th week submitted to feed restriction and received saline injections; ***CRB*** (*n* = 7), fed control diet ad libitum during 9 weeks and during the 10th week submitted to feed restriction and received butaphosphan injections; ***HRS*** (*n* = 7)*,* fed HCD ad libitum for 9 weeks and during the 10th week were submitted to feed restriction and received saline injections; and ***HRB*** (*n* = 7), fed HCD ad libitum for 9 weeks and during the 10th week were submitted to feed restriction and received butaphosphan injections.

### 2.2. Butaphosphan Treatment and Nutritional Management

**Figure 1:**
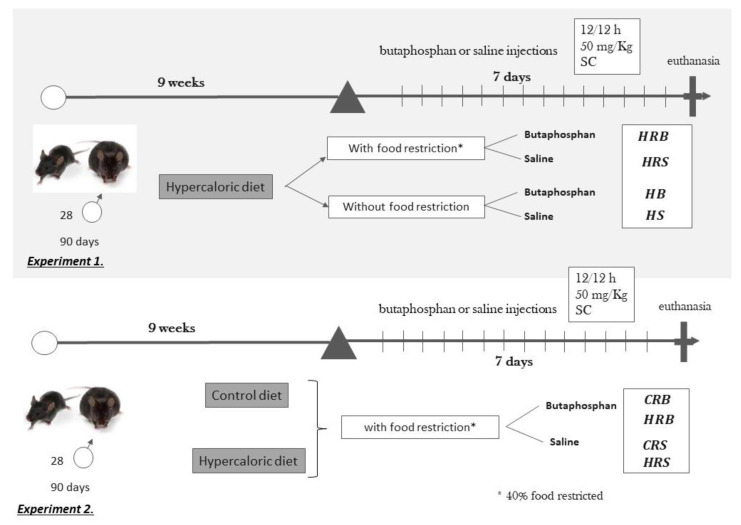
Experimental design. Experiment 1: all mice received hypercaloric diet ad libitum for 9 weeks and then, during the 10th week were randomized into four groups with or without food restriction and with butaphosphan or saline injections, each 12 h. Experiment 2: mice were initially randomized to hypercaloric or control diet for 9 weeks, and during the 10th week, all of them were submitted to food restriction and were treated with butaphosphan or saline injections each 12 h.

For both experiments, at the end of the 9th week, butaphosphan (50 mg/kg, twice a day, 7 days) or saline treatment started (Figure 1). Saline or butaphosphan injections were performed subcutaneously, using an insulin syringe. All groups had their food intake and body weight recorded weekly. Restriction groups (HRS; HRB; CRS; and CRB), during the 10th experimental week received 60% of the amount of food consumed in the previous week.

### 2.3. Diet

Prior to this experiment, C57BL/6 male mice were fed with a pelletized commercial diet (Nuvilab^®^, Nuvital, Brazil) until 90 days old. 

Control diet was the same as commercial diet (Nuvilab^®^, Nuvital, Brazil), with 28% crude protein (CP), 5% fat, 49% non-fiber carbohydrates (NFC), and estimated crude energy (CE) of 3610.5 Kcal/kg. HCD were prepared with 68% Nuvilab^®^ diet (Nuvital, Colombo, Brazil), 26% of sweetened condensed milk, 1% corn starch, 5% vegetable oil, and 2.5% water. This diet was molded into pellets and then dried at 50 °C for 4 h. Composition was 27.8% CP, 14% fat, 68.5% NFC, and CE 4681.7 Kcal/kg. HCD was prepared every two days, in order to prevent fungi development.

### 2.4. Samples Collection

At the end of the 10th week, all mice (Experiment 1 and Experiment 2) were anesthetized with halothane (TanoHalo, Crisalia^®^, Brazil) and euthanized by decapitation. Blood was taken directly from the inferior vena cava and then centrifuged at 3000× *g* for 15 min. at 4 °C for serum isolation, and then stored at −80 °C until analysis. Liver and epididymal white adipose tissues were harvested, weighted, snap-frozen in liquid nitrogen, and stored at −80 °C for further analysis.

### 2.5. Blood Biochemical Analysis

Serum glucose, NEFA, phosphorus, and insulin concentrations were analyzed. NEFA were analyzed by colorimetric assay using a commercial kit (Wako NEFA HR kit; Wako chemicals, Richmond, VA, USA) according to protocol described by Ballou et al. [23] and read on a microplate reader (Thermo Plate, São Paulo, Brazil). For insulin analysis, the commercial ELISA Rat/Mouse insulin ELISA Kit (Merck Millipore, Darmstadt, Germany) was used, and procedures followed the manufacturer’s instructions. Glucose and phosphorus concentrations were measured using commercially available tests (Labtest diagnóstica, Lagoa Santa, Brazil), in an automatic colorimetric analyzer Labmax Plenno (Labtest diagnóstica, Lagoa Santa, Brazil). Based on glucose and insulin results, the HOMA index was calculated [24] using the software HOMA 2 calculator 2.2.3 (Diabetes Trials Unit, University of Oxford), available at www.dtu.ox.ac.uk/homacalculator.

### 2.6. Gene Expression Analysis

Total liver RNA was extracted using TRIzol reagent (Invitrogen, Carlsbad, CA, USA) followed by a purification step (MiRNEasy mini Kit, Qiagen, Hilden, Germany) and treated with RNase (RNase Free DNAse Set, Qiagen, Germany), according to the manufacturer’s recommendations. RNA concentration was measured using Nanodrop Lite spectrophotometer (Thermo Fischer Scientific Inc., Waltham, MA, USA). The A260/A280 ratio was used as an indicative of sample quality and the integrity of the extracted RNA was observed through agarose electrophoresis (80 V, for 1.5 h).

Complementary DNA (cDNA) was performed using i-script cDNA synthesis kit (Bio-Rad, Hercules, CA, USA). The reaction was performed in MyCycle™ Thermo Cycler (Bio-Rad Laboratories, Hercules, CA, USA) using the following temperatures: 25 °C for 10 min, 37 °C for 120 min, and 85 °C for 5 min. The cDNA was diluted to 5 ng/μL. Quantitative polymerase chain reaction (qPCR) with SYBR green was used to evaluate the expression of genes related to insulin signaling, glycolysis, gluconeogenesis, and fatty acid metabolism pathways, using previously tested primers, as shown in the supplemental material (Table S1). Primer sequences were blasted using Blastn against genomic mouse and transcript sequences to ensure specificity. β actin was used as a housekeeping gene.

The q-PCR reaction was composed of 4 μL of the previously diluted cDNA (5 ng/μL), 5 μL of Sybr Green PCR Master Mix (Thermo Fischer Scientific Inc., Waltham, MA, USA), 0.4 μL of each forward and reverse primer, and 0.2 μL nuclease free water (Merck Millipore corporation, Germany), which were placed in 48-well microplates (Bio-Rad Laboratories, Hercules, CA, USA), ran in duplicate with a negative control on each plate. Reactions were performed in ECO Thermocycler Real-Time PCR System (Illumina^®^, San Diego, CA, USA) using the following protocol: incubation period of 2 min at 50 °C, activation of the polymerase at 95 °C for 10 min, followed by 45 cycles of 10 s at 95 °C, 30 s at 60 °C, and a melting stage (15 s at 95 °C, 15 s at 55 °C, and 15 s at 95 °C).

The qPCR results were analyzed in the LinReg PCR software [25], through which the efficiency of the PCR for each plate was calculated. Data were normalized with β actin expression and presented as relative expression.

### 2.7. Statistical Analysis

Statistical analyses were performed using mixed model of SAS 9.4 (SAS Institute Inc., Cary, NC). For experiment 1, fixed effects were treatment (control or butaphosphan), restriction (with or without) and their interaction. For experiment 2, fixed effects were treatment (control or butaphosphan), diet (control or hypercaloric), and their interaction. In both experiments, animal was considered as a random effect. Results are expressed by means ± standard error of the mean (*SEM*). Values of *p* < 0.05 were considered significant and *p* < 0.1 tendency.

## 3. Results

### 3.1. Experiment 1

Total body weight and liver weight were not affected (*p* > 0.05) by butaphosphan treatment or food restriction during the experiment. It was observed a treatment x restriction interaction (*p* = 0.019) on white adipose tissue (WAT mass), mainly due to a treatment effect, and it was observed that mice receiving butaphosphan preserved WAT mass when compared to saline ones (*p* = 0.047). Further, a treatment x restriction interaction (*p* = 0.016) on muscle weight was observed; however, this was mainly due to a restriction effect (*p* = 0.018), which was much more evident on mice treated with saline injections, where food restriction resulted in lower muscle weight (Table 1).

Regarding blood biomarkers, glucose and the HOMA index had interaction, treatment, and restriction effects. Butaphosphan treated mice had higher (*p* < 0.001) glucose concentration compared to the saline ones, but no restriction effect was observed. Lower glucose concentration was observed on restricted mice that received saline injections (*p* < 0.001). The same behavior was observed on the HOMA index, butaphosphan increased (*p* < 0.015) and food restriction decreased (*p* < 0.015) it; however, this reduction was only observed in animals which did not receive butaphosphan injections. It was observed a treatment × restriction interaction on NEFA (*p* = 0.009). Food restriction tended to reduce NEFA (*p* = 0.066) in mice treated with saline injections and tended to increase when mice were treated with butaphosphan (*p* = 0.087). Butaphosphan treatment increased NEFA concentration in mice with food restriction compared with the saline ones (*p* = 0.0022), but when there was no food restriction, butaphosphan did not affect NEFA concentration (*p* = 0.88). Insulin and phosphorus concentration did not differ between groups (Table 1).

Among all evaluated genes, only *Gck* presented a treatment restriction interaction effect (*p* = 0.005). Mice treated with butaphosphan and submitted to food restriction had higher *Gck* hepatic expression (*p* = 0.005); however, it did not differ in the saline ones (*p* = 0.87). In mice with no food restriction, butaphosphan reduced *Gck* (*p =* 0.02) expression. Butaphosphan treatment increased mRNA expression of *Acox1* (*p* = 0.011). When mice were not submitted to food restriction, butaphosphan increased *Acox 1* (*p* = 0.03), but no effect was observed on mice under food restriction (*p* = 0.74). Food restriction reduced (*p* < 0.05) *G6Pase* and *Irs1* mRNA expression, while increased (*p* < 0.001) *Gck* and tended to increase *Acox1* (*p* = 0.089), *Pck 1*(*p* = 0.059), and *Cpt1a* (*p* = 0.071) (Table 2).

### 3.2. Experiment 2

There was no difference in food consumption (control vs. hypercaloric: 33.46 ± 1.67 g/day vs. 36.45 ± 0.77 g/day; *p* = 0.3), but mice fed hypercaloric diet consumed more calories than those in control diet (156.65 ± 7.82 Kcal vs. 131.60 ± 2.78 Kcal, *p* = 0.005), which reflected in increased body mass (*p* = 0.001) and WAT mass (*p* = 0.001). Not only the hypercaloric diet, but also butaphosphan preserved WAT mass (*p* = 0.019) (Table 3).

There was a diet treatment interaction on glucose concentration (*p* = 0.011), butaphosphan treatment increased (*p* = 0.002) blood glucose; however, no diet effect was observed (*p* = 0.297). The HOMA index was increased by butaphosphan treatment (1.63 ± 0.26 vs. 2.36 ± 0.23; *p* = 0.027). Insulin, phosphorus, and NEFA concentration were not altered in this experiment (*p* > 0.05, Table 3).

Regarding gene expression results, there was a treatment x diet interaction (*p* = 0.028) on *Irs2*, with a marked treatment effect (*p* = 0.003), where butaphosphan treatment increased mRNA expression of insulin receptor substrate 2. *Irs1* hepatic mRNA expression was also increased due to a butaphosphan treatment (*p* = 0.002) and reduced (*p* < 0.001) on mice fed hypercaloric diet. *Irs1:Irs2* ratio had a treatment diet interaction effect (*p* = 0.035), mainly due to a diet effect (*p* < 0.001), as animals fed with HCD had lower ratio (Table 4).

Butaphosphan treatment increased (*p* = 0.059) mRNA expression of peroxisome proliferative activated receptor coactivator 1 alpha (*Ppargc1a*). When mice received the hypercaloric diet, butaphosphan did not impact *Ppargc1a* mRNA expression (*p* = 0.27), but it tended to increase when mice received the control diet (*p* = 0.07) (Table 4).

In a situation with food restriction, the hypercaloric diet increased glucokinase (*Gck*, *p* = 0.020) and phosphatidylinositol 3-kinase (*PI3K*, *p* = 0.038) mRNA expression (Table 4).

When evaluated in an isolated form, all genes with a treatment effect increased (*p* < 0.05 for all) the expression when butaphosphan injections were received (*Acaca*, *Pparg*, *PI3K*, *Acox1*, *Ppargc1a*, *Gck*, *Cpt1a*, *Irs1 and Irs2*) (Table 4).

## 4. Discussion

We have investigated the hypothesis that butaphosphan could increase serum glucose in a situation of caloric restriction due to an increase in peripheral insulin resistance. Our results showed that butaphosphan preserved WAT mass, increased serum glucose, and increased the HOMA index. As expected, hypercaloric diet also increased WAT mass and body mass, and finally food restriction reduced the weight of the gastrocnemius muscle and reduced glucose concentration and the HOMA index.

In the present study, it was demonstrated that butaphosphan increased serum glucose concentration in mice, which is in accordance with previous studies that used the association of butaphosphan and cyanocobalamin in cattle and ewes during the periparturient period [5,10,13], where authors suggested that these results were mainly related to cyanocobalamin action, a cofactor for methyl malonyl CoA mutase, which catalyzes the conversion of propionate to succinyl-CoA, a key factor to generate energy through gluconeogenesis and the Krebs Cycle. However, the present study demonstrated that butaphosphan, as an isolated compound, has an important effect on the energy metabolism in mice.

Phosphorus has an important function in carbohydrate hepatic metabolism, once intermediates in the gluconeogenic pathway must be phosphorylated [26]. So, glycolytic and gluconeogenic pathways are both regulated by phosphorus bioavailability. This may explain the effect of butaphosphan (an organic phosphorus compound) on glucose metabolism, increasing glucose concentration. The same results were observed by Furll et al. [5] who reported that multiple intravenous injections of butaphosphan associated with cyanocobalamin before parturition increased glucose availability.

Although we have not evaluated glucagon concentrations, Nuber et al. [9] working with early lactating dairy cows, demonstrated that the administration of butaphosphan increased glucagon when compared to those cows in the control group or receiving butaphosphan in combination with cyanocobalamin. Glucagon is a pancreatic hormone that improves carbohydrate status by stimulating hepatic gluconeogenesis, glycogenolysis, amino acid uptake, and ureagenesis [27,28]. Glucagon is known to increase lipolysis and ketogenesis, but its effect is restrained by increased insulin concentrations [29]. It was suggested that reducing glucagon action or its secretion will lead to potent reductions in the elevated hepatic glucose production observed in both Type I and II diabetes mellitus [30]. A recent study showed that Ppargc1a influences the balance between IRS1 and IRS2 expression in liver cells, and as a result, the coactivator plays a key role in insulin-mediated control of gluconeogenesis during the fasting-to-fed transition [31]. So, the increase in glucose concentrations, Ppargc1a expression, and also the Irs1:Irs2 ratio observed in our study, could be coupled with an increase in glucagon concentrations. However, glucagon concentration was not evaluated in our study, and needs to be confirmed.

The main effects of butaphosphan observed in the present study in mice submitted to caloric restriction were the preservation of the adipose tissue mass, an increased blood glucose and the HOMA index, without a difference in insulin concentrations. Indeed, when mice were fed a control diet and submitted to a caloric restriction or fed an hypercaloric diet but with no caloric restriction, butaphosphan supplementation did not affect the blood parameters, confirming that the butaphosphan effects are more evident in acute depletion of the energy status in mice. The same results were reported by other studies that have shown that the effect of butaphosphan and cyanocobalamin in increasing milk production in cattle is more pronounced when animals are in an acute state of negative energy balance, mainly in overweight animals with higher WAT reserves [32]. Our results suggest that the effects of organic phosphorous supplementation involve changes in mechanisms related to glucose homeostasis and the insulin signaling process when there is a reduction in the energy status, such as that observed in dairy cattle experiments.

The results demonstrated the potential action of butaphosphan in reducing fatty acid accumulation in the liver of animals that have undergone a diet alteration, since animals treated with butaphosphan had higher expression of genes related to fatty acid metabolism such as *Acox1*, *Cpt1a,* and *Acaca*. Additionally, butaphosphan did not increase serum phosphorus concentration once phosphorus was involved in cholesterol metabolism and fatty acids biosynthesis. Tanaka et al. [15] reported that a phosphorus restriction increased steatosis in rats receiving a diet rich in cholesterol, and this restriction decreased the expression of genes linked to cholesterol metabolism and fatty acids biosynthesis.

*Acox1* expression is directly related to *Cpt1a* expression, as it is a rate-limiting enzyme that catalyzes the first step of long-chain fatty acid oxidation, and any disturbance in the expression or activity of this enzyme leads to the development of steatohepatitis [33]. Orellana-Gavaldà, et al. [34] showed that mice fed with a diet rich in lipids had higher expression of Cpt1a, increased β oxidation process and did not present steatosis. In our study, liver weight did not differ between mice fed hypercaloric diet and those submitted to food restriction, showing that butaphosphan may be involved in preventing lipid accumulation in the liver. Further studies should include histological analysis to confirm this hypothesis.

Previous reports suggest that in cattle, injections of butaphosphan without the addition of cyanocobalamin were less efficacious in changing plasma concentrations of NEFA and beta-hydroxybutyrate (BHB), and ineffective in changing mRNA expression of genes related to insulin signaling or lipid metabolism [9]. The greater changes observed in our study are probably related to the higher dose of phosphorus applied (50 mg/kg), which is five to ten times greater than studies with cattle. According to manufacturer, the recommended dose for cattle is 5 mg/kg, and in the study conducted by [9], they used a dose of 10 mg/kg.

According to Farese Jr et al. [35], lipids can impair hepatic cell function, especially on their capacity to respond to changes on insulin concentration, which is in accordance with our results where we demonstrated that butaphosphan increased blood NEFA concentration in mice receiving hypercaloric diet and submitted to food restriction, coupled with an increase in the HOMA index. Margolis et al. [36] demonstrated that rats submitted to feed restriction had a lower HOMA index when compared to ad libitum feeding, which was also associated with a reduction in insulin resistance. It was interesting that mice fed the hypercaloric diet and submitted to caloric restriction and supplemented with butaphosphan, increased the HOMA index when compared to the saline group, indicating again that butaphosphan effect depends on insulin signaling. According to Menon et al. [24], the HOMA index estimates the hepatic insulin sensibility, where lower values are indicative of higher hepatic insulin sensibility. So, in this case, butaphosphan reduced hepatic insulin sensibility on mice receiving hypercaloric diet and submitted to restriction.

A research conducted with no lactating and no pregnant cattle fed with hypercaloric diet showed that an increased in NEFA reduced the response capacity of insulin receptors to glucose concentration [37,38]. This impaired capacity of hepatocytes leads to glucose intolerance and, consequently, hyperglycemia and type II diabetes [35]. We observed that, as butaphosphan increased the HOMA index in mice with the hypercaloric diet and submitted to restriction, NEFA also tended to increase.

To confirm the alterations on insulin signaling pathway, and to study the glucose and lipid metabolism changes associated to butaphosphan supplementation, the hepatic gene expression of some key genes in the regulation of energy metabolism were analyzed. Butaphosphan increased *Gck* mRNA expression. Since Gck are regulated by insulin, these data are in accordance with the hypothesis that the butaphosphan effect depends on insulin signaling. At hepatic level, insulin resistance increases glucose production is due to an impaired ability of insulin to suppress the activity of gluconeogenic enzymes [39]. Increased hepatic insulin resistance is associated with hyperinsulinemia, which consequently increases triacylglycerol acylation and reduces lipolysis in adipose tissue, where fat mobilization is strongly inhibited by insulin [40].

*Ppargc1a* plays a central role in the regulation of cellular energy metabolism. It links environmental stimulus to adaptive thermogenesis, stimulates mitochondrial biogenesis, and promotes the remodeling of muscle tissue to a fiber-type composition that is naturally more oxidative and less glycolytic. It also participates in the regulation of both carbohydrate and lipid metabolism [41,42,43]. According to Puigserver et al. [44], *Pargc1a* requires *FoxO1* to bind and localize to the promoter region of gluconeogenic genes. So far, *HNF4α* and *FoxO1* are key transcription factors required for *Pargc1a* induction of gluconeogenic genes. In this study, besides having observed a tendency for *Ppargc1a* to increase in mice receiving butaphosphan, treatment did not alter *FoxO1*, suggesting that there was no influence on gluconeogenesis. However, *Ppagc1a* also activates other metabolic pathways such as fatty acid oxidation. The phosphorylation of Akt can induce the translocation of GLUT4 from the cytoplasm to the plasma membrane, which promotes the transport of glucose into the cell [45]. Zhu et al. [46] demonstrated that the improvement of glucose uptake in insulin-resistant adipocytes was dependent on the PI3K/Akt pathway.

Under normal energy balance, *Ppargc1a* is expressed at very low levels in the liver [44,47]. However, fasting produces a robust increase of *Ppargc1a* expression, which, in turn, stimulates hepatic gluconeogenesis and fatty acid oxidative metabolism. In the present study, mice receiving a hypercaloric diet showed increased *Ppargc1a* expression, and butaphosphan also increased the mRNA expression, indicating that butaphosphan interferes with fatty acid metabolism. Low expression of this gene is related to insulin resistance by interfering lipid oxidation, oxidative stress, and it acts on the regulation of pancreatic insulin secretion. In accordance, human with Type II diabetes presented reduced expression of Ppargc1a [45,48]. The increase of *Ppargc1a* expression observed in mice treated with butaphosphan, when receiving a control diet and submitted to a caloric restriction, and no differences in mRNA expression when butaphosphan was associated with hypercaloric diet and food restriction, strengthens the hypothesis that the action of butaphosphan is associated with insulin resistance in an acute energy deficit situation.

For the first time the relationship of butaphosphan with energy metabolism and insulin signaling was demonstrated, observed in mice at hepatic level. These promising results open the field to investigate the potentials of this molecule in treatment of metabolic syndrome. Certainly, complementary biomarkers and tissues should be evaluated in further studies.

## 5. Conclusions

The results of this study indicate that butaphosphan treatment increased glucose and preserved WAT in mice submitted to a caloric restriction, and that these effects seem to be mediated by insulin signaling, which is much more evident when animals are fed a hypercaloric diet. Further investigations are required to deeply understand the effects of butaphosphan on insulin signaling regulation of different tissues and species.

## Figures and Tables

**Table 1:** Tissue weight and blood biomarkers of mice fed a hypercaloric diet, treated with butaphosphan or saline solution, and submitted or not to food restriction (Experiment 1).

	Groups					*p*-Value		
	HB	HRB	HS	HRS	*SEM*	Treat	Restriction	Treat. × Restriction
^a^ Body weight (g)	33.28	34.71	34.85	32.2	1.76	0.766	0.699	0.205
^b^ Liver (g)	1.57	1.73	1.74	1.77	0.10	0.237	0.299	0.492
^c^ Epididymal WAT mass (g)	0.88 ^a,b^	1.11 ^a^	0.95 ^a,b^	0.36 ^b^	0.18	0.047	0.284	0.019
^d^ Muscle (g)	0.18 ^a,b^	0.18 ^a,b^	0.19 ^a^	0.14 ^b^	0.01	0.324	0.018	0.016
^e^ Glucose (mmol/L)	12.61 ^a^	11.35 ^a^	11.25 ^a^	7.51 ^b^	0.48	<0.001	<0.001	0.008
^f^ NEFA (mmol/L)	0.80 ^ab^	0.85 ^a^	0.85 ^a^	0.65 ^b^	0.05	0.066	0.087	0.009
^g^ Insulin (ng/mL)	0.29	0.32	0.31	0.27	0.03	0.539	0.488	0.284
^h^ Phosphorus (mg/dL)	10.14	9.28	10.14	9.94	0.69	0.125	0.925	0.672
^I^ HOMA Index	2.96 ^a^	2.91 ^a^	2.82 ^a^	1.73 ^b^	0.36	0.015	0.015	0.045

Body weight corresponds to absolute body weight at the end of the experiment; (^b^) corresponds to the liver weight after euthanasia; (^c^) WAT mass is relative to epididymal white adipose tissue depots after euthanasia; (^d^) Gastrocnemius muscle; (^e^) serum level of glucose; (^f^) serum level of non-esterified fatty acids; (^g^) insulin serum concentration; (^h^) phosphorus serum concentration; and (^i^) HOMA Index based on glucose and insulin concentration. Different letters represent statistical difference between groups (*p <* 0.05).

**Table 2:** Hepatic mRNA relative expression of mice fed hypercaloric diet, treated with butaphosphan or saline solution, and submitted or not to food restriction (Experiment 1).

Gene	Groups					*p*-Value		
	HB	HRB	HS	HRS	*SEM*	Treat.	Restriction	Treat. × Restriction
*Lipid metabolism*								
*Acaca*	0.36	0.25	0.15	0.18	0.09	0.098	0.302	0.385
*Acox1*	2.16	2.45	0.74	1.33	0.57	0.011	0.089	0.201
*Cpt1a*	0.82	1.11	0.80	1.03	0.14	0.672	0.071	0.995
*Srebp1c*	0.24	0.08	0.10	0.09	0.08	0.384	0.626	0.248
*Glucose metabolism and insulin signaling*								
*PcK1*	0.73	0.36	0.61	0.45	0.21	0.824	0.059	0.928
*GcK*	0.11 ^b^	0.39 ^a^	0.45 ^a^	0.18 ^a,b^	0.15	0.217	0.050	0.005
*Fbp1*	2.07	3.01	1.87	2.39	0.92	0.316	0.122	0.241
*G6Pase*	0.45	0.10	0.43	0.09	0.11	0.819	0.0002	0.921
*PI3K*	0.16	0.15	0.10	0.11	0.05	0.340	0.400	0.617
*Irs1*	0.58	0.19	0.55	0.10	0.10	0.135	<0.0001	0.170
*Irs2*	0.12	0.18	0.18	0.14	0.05	0.460	0.164	0.162
*Irs1:Irs2*	6.30	1.17	3.58	0.72	0.97	0.051	<0.001	0.971
*FoxO1*	0.24	0.25	0.13	0.12	0.11	0.124	0.517	0.757
*Transcription Factors*								
*Pparγ*	0.031	0.026	0.028	0.014	0.001	0.223	0.577	0.593
*Ppargc1a*	0.006	0.03	0.005	0.04	0.007	0.783	<0.0001	0.274

Different letters represent statistical difference between groups (*p* < 0.05). *SEM* represents the highest standard error of the mean.

**Table 3:** Physical and blood biomarkers of mice receiving different treatments (butaphosphan or saline), control or hypercaloric diet, and food restricted (Experiment 2).

	Groups					*p*-Value		
	CRB	HRB	CRS	HRS	*SEM*	Treat.	Diet	Treat. × Diet
^a^ Body Mass (g)	29.43	34.71	28.71	32.20	1.29	0.175	0.001	0.442
^b^ Liver (g)	1.62	1.73	1.61	1.77	0.09	0.832	0.132	0.744
^c^ WAT mass (g)	0.21 ^b^	1.10 ^a^	0.24 ^b^	0.36 ^b^	0.15	0.019	0.001	0.011
^d^ Muscle (g)	0.15	0.18	0.15	0.14	0.01	0.063	0.485	0.068
^e^ Glucose (mmol/L)	9.02 ^b^	11.35 ^a^	8.54 ^b^	7.5 ^b^	0.68	0.002	0.297	0.011
^f^ NEFA (mmol/L)	0.84	0.85	0.91	0.65	0.1	0.54	0.39	0.53
^g^ Insulin (ng/mL)	0.25	0.32	0.22	0.27	0.04	0.336	0.119	0.644
^h^ Phosphorus (mg/dL)	11.06	9.28	9.56	9.94	0.69	0.453	0.236	0.118
^I^ HOMA Index	1.80	2.91	1.53	1.73	0.39	0.027	0.115	0.125

(^a^) Body Mass corresponds to absolute body weight at the end of the experiment; (^b^) corresponds to the liver weight after euthanasia; (^c^) WAT mass is relative to abdominal white adipose tissue depots after euthanasia; ^d^) Gastrocnemius muscle; (^e^) serum level of glucose; (^f^) serum level of non-esterified fatty acids; (^g^) insulin serum concentration; (^h^) Phosphorus serum concentration; and (^i^) HOMA Index based on glucose and insulin concentration. Different letters represent statistical difference between groups (*p* < 0.05).

**Table 4:** Hepatic mRNA relative expression of mice receiving different treatments (butaphosphan or saline), control or hypercaloric diet, and food restricted (Experiment 2).

Gene	Groups					*p*-Value		
	CRB	HRB	CRS	HRS	SEM	Treat.	Diet	Treat. ×Diet
*Lipid metabolism*								
*Acaca*	0.23	0.25	0.13	0.18	0.04	0.055	0.120	0.538
*Acox1*	1.55	2.45	0.98	1.33	0.52	0.026	0.113	0.726
*Cpt1a*	1.32	1.11	0.84	1.02	0.14	0.034	0.844	0.100
*Srebp1c*	0.07	0.08	0.06	0.10	0.01	0.822	0.110	0.724
*Glucose metabolism and insulin signaling*								
*PcK1*	0.29	0.36	0.47	0.45	0.11	0.287	0.629	0.223
*GcK*	0.25	0.39	0.12	0.18	0.06	0.014	0.020	0.839
*Fbp1*	3.29	3.01	2.12	2.39	0.69	0.332	0.603	0.478
*G6Pase*	0.14	0.11	0.19	0.09	0.04	0.551	0.142	0.375
*PI3K*	0.11	0.15	0.04	0.11	0.03	0.030	0.038	0.124
*Irs1*	0.56	0.19 *	0.33	0.10 *	0.06	0.002	<0.0001	0.840
*Irs2*	0.21 ^a^	0.18 ^a^	0.09 ^b,^*	0.14 ^a,b,^*	0.02	0.003	0.202	0.028
*Irs1:Irs2*	2.72 ^a^	1.17 ^b^	5.20 ^a^	0.72 ^b^	0.98	0.793	<0.001	0.035
*FoxO1*	0.15	0.25	0.10	0.12	0.07	0.272	0.300	0.939
*Transcription Factors*								
*Pparγ*	0.02 *	0.02	0.01 *	0.01	0.005	0.005	0.278	0.710
*Ppargc1a*	0.04 ^a^	0.03 ^a^	0.01 ^b^	0.04 ^a^	0.009	0.059	0.27	0.002

Different letters represent statistical difference between groups (*p* < 0.05). Asterisk (*) represents a tendency to means differ between groups (0.05 < *p* < 0.09). *SEM* represents the highest standard error of the mean.

## Supplementary Materials

The following are available online at https://www.mdpi.com/2072-6643/12/6/1856/s1, Table S1: Primers sequence for qRT PCR.
