# Supplementary material for: Butaphosphan Effects on Glucose Metabolism Involve Insulin Signaling and Depends on Nutritional Plan
Source: Nutrients. 2020 Jun 22;12(6):1856. doi: 10.3390/nu12061856 (PMC7353219; doi:10.3390/nu12061856)
Supplement: Supplementary file 1 [file nutrients-12-01856-s001.pdf]

Table S1. Primers sequence for qRT PCR.

Gene	Official name	Forward (5`→ 3`)	Reverse (5 → 3`)
FoxO1	Forkhead box O1	GCTTTTGTCACGATGGAGGT	CGCACAGAGCACTCCATAAA
Irs1	Insulin receptor substrate 1	ATGGCGAGAGCCCTCCGGATACC	CTCATAATACTCCAGGCGCGC
Irs2	Insulin receptor substrate 2	GCCCGAACCTCAATAACAAC	CTTGTGGCCGTGCTTCTG
PI3K	Phosphatidylinositol 3-kinase	TAGCTGCATTGGAGCTCCTT	TACGAACTGTGGGAGCAGAT
Ppargc1a	Peroxisome proliferative activated receptor, gamma, coactivator 1 alpha	GTCAACAGCAAAAGCCACAA	TCTGGGGTCAGAGGAAGAGA
Pck1	Phosphoenolpyruvate carboxykinase 1	GACAGCCTGCCCCAGGCAGTGA	CTGGCCACATCTCGAGGGTCAG
Gck	Glucokinase	GAGATGGATGTGGTGGCAAT	ACCAGCTCCACATTCTGCAT
Fbp1	Fructose bisphosphatase 1	GACCCTGCCATCAATGAGTA	GTTGGCGGGGTATAAAAAGA
G6pc	Glucose-6-phosphatase	TGCTGCTCACTTTCCCCACCAG	TCTCCAAAGTCCACAGGAGGT
Acaca	Acetyl-Coenzyme A carboxylase	GGACAGACTGATCGCAGAGAAAG	GCTGTTCCTCAGGCTCACAT
Acox1	Acyl-Coenzyme A oxidase 1	GTGCAGCTCAGAGTCTGTCCAA	TACTGCTGCGTCTGAAAATCCA
Cpt1a	Carnitine palmytoil transferase 1	CTTCCATGACTCGGCTCTTC	AGCTTGAACCTCTGCTCTGC

*primer sequences were blasted using Blastn.
